# *Dialister pneumosintes* bacteremia associated with sinusitis and suspected meningitis: a case report and literature review

**DOI:** 10.1186/s12879-026-13555-5

**Published:** 2026-05-16

**Authors:** Mari Yamamoto, Toshikazu Ozeki, Hiroki Ikai, Waka Yokoyama-Kokuryo, Mayumi Ishikawa, Seiya Itou, Yuna Hiramatsu, Fumika Nagase, Yuki Itou, Naoho Takizawa, Yoshiro Fujita

**Affiliations:** 1 Department of Rheumatology and Nephrology, Chubu Rosai Hospital, 1-10-6 Komei, Minato-ku, Nagoya, Aichi 455-8530 Japan; 2 Infection Control Team, Chubu Rosai Hospital, 1-10-6 Komei, Minato-ku, Nagoya, Aichi 455-8530 Japan; 3 Scientific Research WorkS Peer Support Group (SRWS-PSG), 1-7-7 Koraibashi, Chuo-ku, Osaka, 541-0043 Japan; 4 Department of Clinical Laboratory, Chubu Rosai Hospital, 1-10-6 Komei, Minato-ku, Nagoya, Aichi 455-8530 Japan

**Keywords:** *Dialister pneumosintes*, Bacteremia, Meningitis, Sinusitis, Anaerobic infection, MALDI-TOF MS, Case report

## Abstract

**Background:**

*Dialister pneumosintes*(*D. pneumosintes*) is a slow-growing, obligate anaerobic Gram-negative bacillus commonly found in the oral cavity, nasopharynx, gastrointestinal tract, and vaginal microbiota. Although typically associated with periodontal disease, *D. pneumosintes* has rarely been implicated in bloodstream infections and, to date, has not been reported as a causative agent of meningitis. This case report describes *D. pneumosintes* bacteremia complicated by meningitis, highlighting the diagnostic challenges associated with fastidious anaerobes and the role of advanced microbiological techniques, including matrix-assisted laser desorption/ionization time-of-flight mass spectrometry (MALDI-TOF MS) and 16S rRNA gene analysis, in accurate pathogen identification.

**Case presentation:**

A 65-year-old woman presented with fever, dysarthria, and posterior neck pain. Clinical examination revealed altered consciousness, restricted neck mobility, bilateral eyelid edema, and nasal speech. Laboratory findings indicated hypokalemia and metabolic alkalosis. Imaging studies revealed marked mucosal thickening and fluid accumulation in the paranasal sinuses, suggesting sinusitis with possible extension to the middle ear. Lumbar puncture before initiation of antibiotic therapy revealed neutrophilic pleocytosis consistent with acute bacterial meningitis. Blood cultures grew *Staphylococcus hominis* and an unidentified anaerobic Gram-negative bacillus, which was later identified as *D. pneumosintes* using MALDI-TOF MS. Empirical antibiotic therapy with ceftriaxone, vancomycin, ampicillin, and corticosteroids was initiated. Persistent fever prompted endoscopic sinus surgery on hospital day 5, with intraoperative cultures yielding *Staphylococcus aureus* and revealing a benign nasal papilloma. Neurological symptoms improved postoperatively. The patient completed 14 days of intravenous antibiotic treatment and was ultimately discharged on day 45.

**Conclusion:**

This is an exceptionally rare case of *D. pneumosintes* bacteremia complicated by meningitis. Given its propensity to originate from odontogenic or sinonasal infections and cause hematogenous dissemination with invasive complications such as abscesses or septic thrombosis, prompt identification is essential. When detected, clinicians should initiate a comprehensive systemic evaluation and consider surgical intervention as part of the treatment strategy. As advanced diagnostic tools such as MALDI-TOF MS and 16S rRNA gene sequencing become more widely available, increased clinical recognition of *D. pneumosintes* as a potential pathogen in severe anaerobic infections is warranted.

**Clinical trial number:**

Not applicable.

## Background

*D. pneumosintes* is a slow-growing, obligate anaerobic Gram-negative bacillus [1] considered a commensal bacterium in the oral cavity, nasopharynx, gastrointestinal tract, and vaginal microbiota [2]. We encountered a case of *D. pneumosintes* bacteremia with clinical features of sinusitis and meningitis. No previous reports have described *D. pneumosintes* infection presenting with meningitis. Accurate identification of fastidious anaerobes such as *D. pneumosintes* often requires advanced diagnostic methods, including MALDI-TOF MS and 16 S rRNA gene analysis, which can support appropriate clinical management of severe infections. We describe our experience with this case, including the diagnosis, treatment course, and patient management.

## Case presentation

A 65-year-old Japanese woman presented to the emergency department with fever and posterior neck pain. She had experienced progressive fatigue and green nasal discharge for 2 months prior to admission. Dysarthria developed 5 days before presentation, followed by a sense of head heaviness and posterior neck pain the day before admission. On the day of presentation, she developed a high-grade fever and impaired mobility, prompting emergency medical transport. She had no significant medical history and had never sought prior hospital or dental care. Multiple untreated dental caries were present. Medications included over-the-counter iron and vitamin supplements, along with herbal remedies such as Hachimijiogan and ginseng. She reported no allergies and had never smoked or consumed alcohol. Her activities of daily living were fully independent, and she lived with two daughters.

The patient worked as a homemaker and had no contact with pets or livestock. On arrival, her vital signs were as follows: temperature 38.5 °C, blood pressure 185/110 mmHg, heart rate 93 bpm, and oxygen saturation 90% on room air. Her height and weight were 155.7 cm and 58.7 kg, respectively. The level of consciousness was mildly impaired (Japan Coma Scale I-3), and she exhibited nasal-sounding dysarthria. Neck examination revealed restricted extension and rotation. Facial findings included bilateral eyelid edema and incomplete eye closure. Conjunctival injection was also noted. Pupils were equal and reactive to light. Oral examination revealed multiple dental caries. Cardiac auscultation revealed no murmurs, and breath sounds were normal. The abdomen was flat and nontender. Bilateral lower leg edema was present. No neurological deficits or limb paralysis were identified. Initial laboratory findings are summarized in Table 1. Inflammatory markers were markedly elevated, supporting a diagnosis of severe infection. Non-contrast head CT and MRI revealed marked mucosal thickening and fluid accumulation in the paranasal sinuses.

**Fig. 1:**
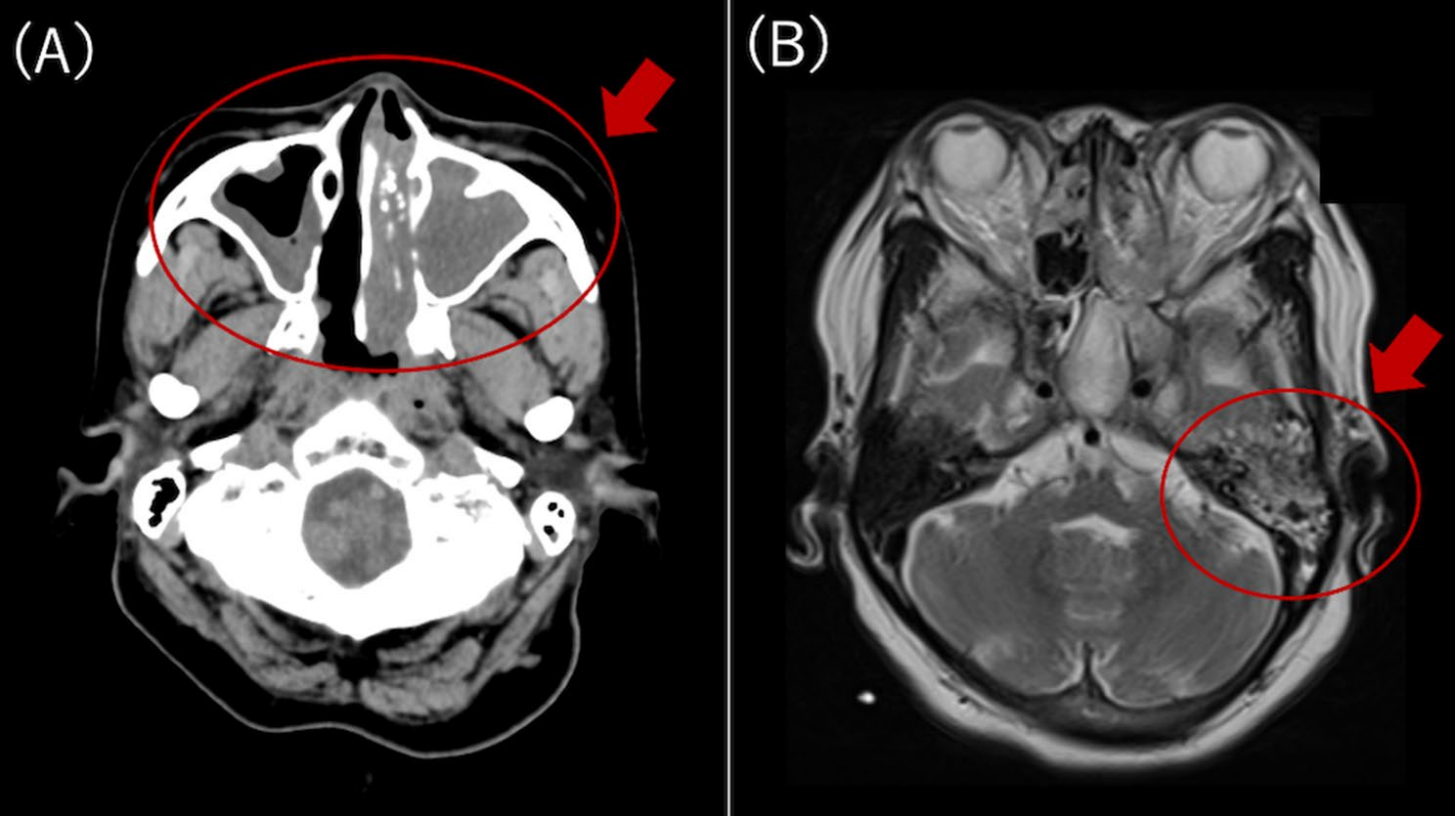
Head computed tomography(CT) and magnetic resonance imaging findings on admission. (**A**) Axial contrast-enhanced CT showing marked mucosal thickening and fluid retention in the paranasal sinuses, predominantly on the left side (red circles). (**B**) Axial T2-weighted MRI demonstrating opacification of the left tympanic cavity and mastoid air cells (red circle), consistent with left-sided otitis media. Red arrows indicate locations of major inflammatory changes

Reduced aeration in the left tympanic cavity and mastoid air cells suggested concurrent otitis media (Fig. 1). A chest-to-pelvis non-contrast CT scan revealed a mass in the left breast suspicious for malignancy. The patient also exhibited hypokalemia and metabolic alkalosis. Given the presence of hypertension and a history of herbal medication use, pseudoaldosteronism was suspected. Subsequent testing confirmed the diagnosis, with serum renin of 0.8 ng/mL and plasma aldosterone concentration < 4.0 pg/mL.

**Table 1:** Laboratory findings on admission

Category	Test	Result	Unit
Hematology	White blood cells	18,800	/µL
	Red blood cells	4.48 × 10⁶	/µL
	Hematocrit	38.5	%
	Mean corpuscular hemoglobin concentration	34.8	g/dL
	Platelet count	17.8 × 10^4^	/µL
Biochemistry	Total protein	7.0	g/dL
	Albumin	2.9	g/dL
	Aspartate aminotransferase	38	U/L
	Alanine aminotransferase	49	U/L
	Lactate dehydrogenase	368	U/L
	Blood urea nitrogen	8.4	mg/dL
	Creatinine	0.63	mg/dL
	Uric acid	5.9	mg/dL
	C-reactive protein	37.98	mg/dL
	Sodium	131	mmol/L
	Potassium	2.2	mmol/L
	Chloride	84	mmol/L
Urinalysis	Specific gravity	1.019	
	pH	6.5	
	Protein	3+	
	Occult blood	3+	
	Leukocytes	-	
	Glucose	-	
	Bacteria	-	

Given the presence of fever, headache, and altered consciousness, bacterial meningitis was suspected, and lumbar puncture was performed before initiation of antibiotic therapy. The cerebrospinal fluid (CSF) appeared slightly turbid with a faint xanthochromic hue. CSF analysis revealed 3,213 cells/µL, with 91% neutrophils and 9% mononuclear cells, consistent with acute bacterial meningitis. Empirical antibiotic therapy was immediately initiated. Only routine aerobic CSF culture was performed, and no anaerobic culture of the CSF specimen was conducted. CSF culture yielded no predominant pathogen. Blood cultures obtained at admission included two sets collected from separate venipuncture sites, each consisting of one aerobic and one anaerobic bottle. *Staphylococcus hominis* and an anaerobic Gram-negative bacillus were isolated from both anaerobic bottles (2 of 4 bottles), whereas aerobic bottles yielded no growth. Matrix-assisted laser desorption/ionization time-of-flight mass spectrometry (MALDI-TOF MS; MALDI Biotyper, Bruker Daltonics, version 13.0.02) identified the anaerobe as *Dialister pneumosintes*, with a score ≥ 2.0, consistent with high-confidence species-level identification according to manufacturer criteria. The exact score value was unavailable because of retrospective data limitations. Based on the clinical course, the patient was urgently admitted to the intensive care unit. Empirical treatment for suspected acute bacterial meningitis was initiated with ceftriaxone (4 g/day), vancomycin (loading dose 20–25 mg/kg followed by 15–20 mg/kg with trough-guided adjustment), ampicillin (12 g/day), and corticosteroids, in accordance with established guidelines [3]. Blood cultures obtained the following day yielded *Staphylococcus hominis* and an anaerobic Gram-negative bacillus, later identified as *Dialister pneumosintes*, and ampicillin was discontinued on hospital day 3.

**Fig. 2:**
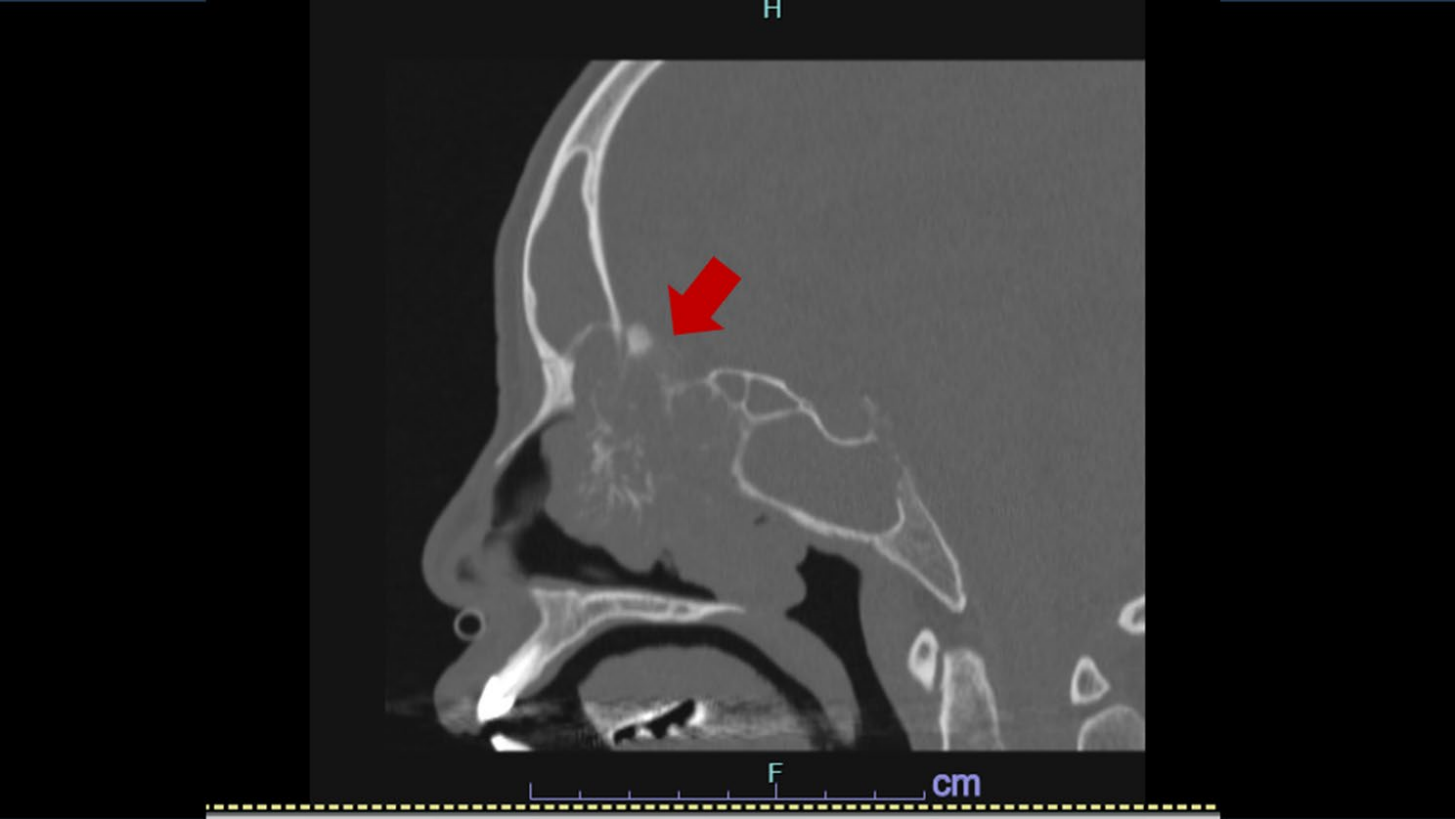
Computed tomography (CT) of the paranasal sinuses. Sagittal CT images demonstrate opacification of the left paranasal sinuses with a mass lesion consistent with a sinonasal papilloma. Partial bone destruction of the sinus wall is observed (arrow), suggesting locally aggressive disease. These findings are consistent with complicated sinusitis with a potential risk of extension beyond the sinus cavity

Despite therapy, the high-grade fever persisted, and endoscopic sinus surgery was performed on hospital day 5. Intraoperative findings demonstrated partial destruction of the paranasal sinus bone caused by the papilloma (Fig. 2). Culture of the sinus specimen yielded methicillin-susceptible *Staphylococcus aureus*. A nasal papilloma was identified in the left nasal cavity.

**Fig. 3:**
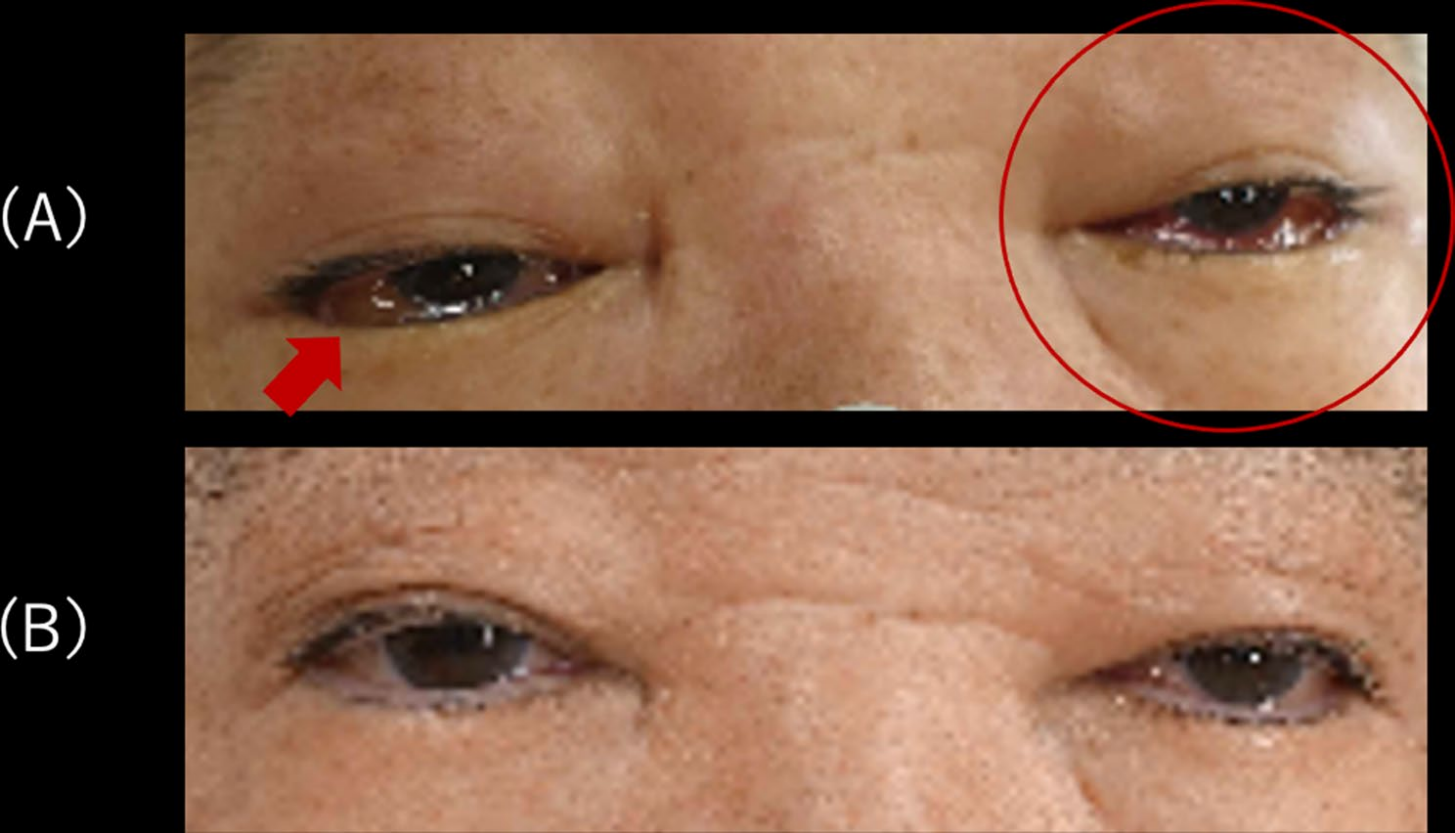
Periorbital findings before and after sinus surgery. (**A**) On day 1, conjunctival chemosis and hyperemia are observed (arrow), along with incomplete eyelid closure and mild eyelid swelling (circle). No purulent ocular discharge or overt signs of ocular infection were observed. These findings were presumed to result from elevated orbital pressure and impaired venous drainage, leading to conjunctival edema. (**B**) By day 7 after sinus surgery, the conjunctival findings had markedly improved, with restoration of eyelid function and resolution of orbital congestion

**Fig. 4:**
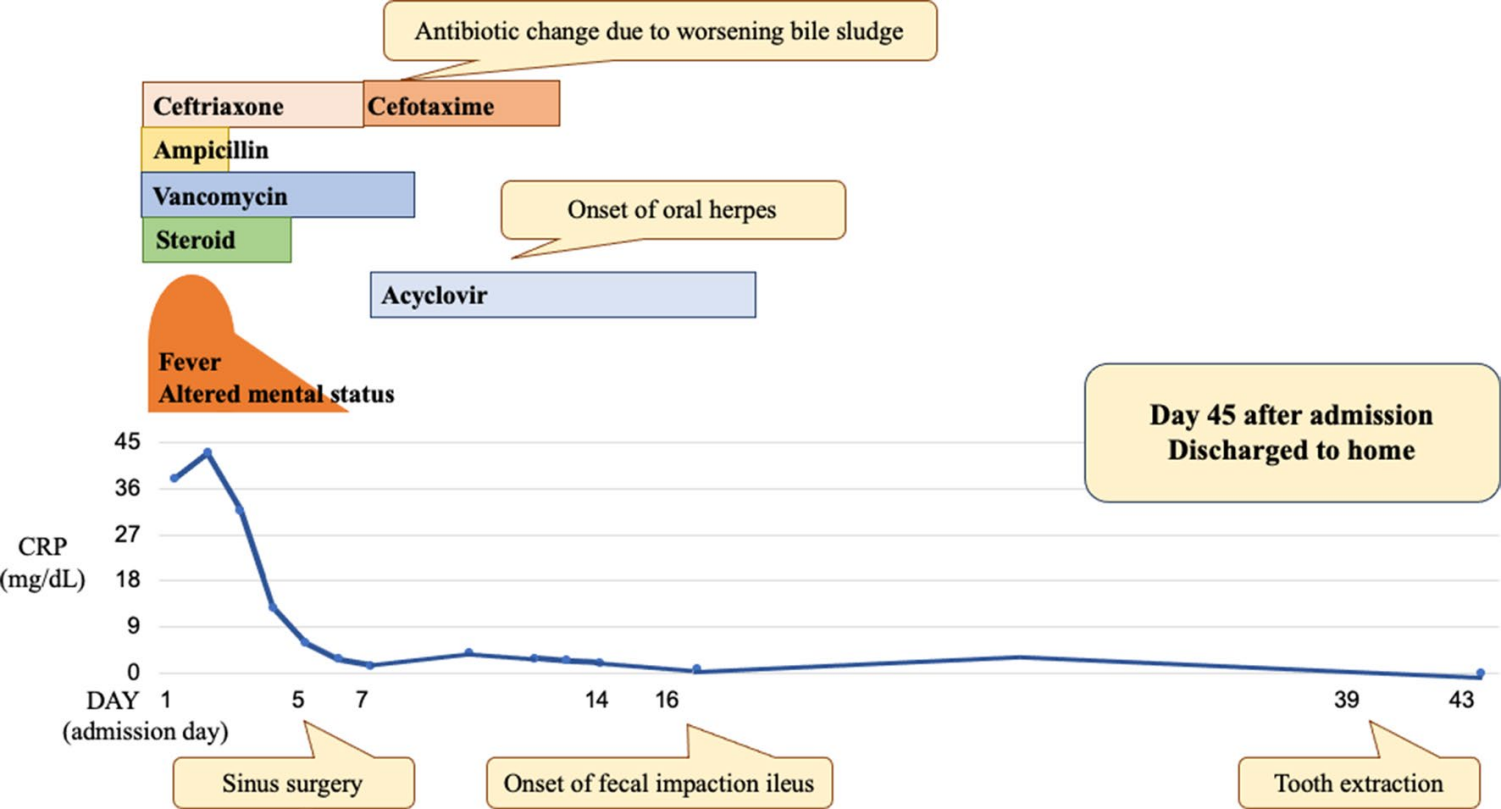
Clinical course, antibiotic regimen, and key interventions. Temporal changes in C-reactive protein (CRP) levels (mg/dL) from admission (day 1) to discharge (day 45) are shown along with antibiotic regimens, steroid use, notable events, and interventions. Sinus surgery was performed on day 5. Fecal impaction ileus was observed on day 14. On day 39, decayed teeth were extracted. Ceftriaxone was switched to cefotaxime because of worsening biliary sludge. An oral herpes outbreak was treated with acyclovir. The patient was discharged on day 45 with a favorable recovery

Histopathological examination did not reveal malignancy. Papilloma-induced obstruction was considered the source of the sinusitis. Eyelid dysfunction and conjunctival injection observed at admission improved beginning the day after surgery (Fig. 3). Orbital compartment syndrome due to increased intraorbital pressure likely caused transient perfusion impairment. Ceftriaxone was switched to cefotaxime on hospital day 7 because of biliary sludge and liver dysfunction and was continued until day 14. Vancomycin was discontinued on hospital day 10 after susceptibility results confirmed no resistant Gram-positive organisms. During hospitalization, the patient developed fecal ileus, which prolonged the hospital stay. Tooth extraction was performed on day 39, and the patient was discharged on day 45 (Fig. 4).

## Discussion and conclusions

*D. pneumosintes* was first isolated in 1921 from the nasopharyngeal secretions of patients during the 1918–1919 influenza pandemic and was initially designated as *Bacterium pneumosintes* [1]. The genus *Dialister* currently includes four recognized species, among which *D. pneumosintes* and *D. micraerophilus* are the most commonly encountered in clinical practice [4]. As a slow-growing obligate anaerobic Gram-negative bacillus, *D. pneumosintes* is difficult to culture using conventional media. Accurate identification requires advanced methods such as 16 S rRNA gene sequencing or mass spectrometry using MALDI-TOF MS [5].

A systematic review and meta-analysis of periodontal pathogens identified *D. pneumosintes* as significantly more prevalent in patients with periodontal disease, particularly in those with advanced periodontitis [6]. This organism exhibits a strong affinity for hypoxic environments, suggesting preferential colonization and proliferation within inflamed periodontal pockets. Beyond the oral cavity, *D. pneumosintes* is also considered a commensal organism in the nasopharynx, gastrointestinal tract, and vaginal microbiota [2]. The patient had poor oral hygiene with untreated dental caries requiring extraction, supporting the oral cavity as a potential reservoir.

Multiple pathogens were identified during the clinical course, including *D. pneumosintes*, *Staphylococcus hominis*, and methicillin-susceptible *Staphylococcus aureus*, with discordant results between blood, sinus, and cerebrospinal fluid (CSF) cultures. This polymicrobial pattern is consistent with the microbiology of acute apical abscesses, in which a mixture of obligate and facultative anaerobes is found in 59–75% of cases [7]. Both *D. pneumosintes* and *S. hominis* were isolated from anaerobic bottles in two separate blood culture sets, supporting true bacteremia rather than contamination. Unlike coagulase-negative staphylococci, *D. pneumosintes* is not typically regarded as a contaminant and has been reported as a pathogen in invasive infections. Its commensal presence in the oral cavity and nasopharynx further supports odontogenic infection or sinusitis as the likely source of bacteremia.

The clinical course suggested the initial development of sinusitis approximately 2 months before admission, followed by an acute onset of dysarthria, headache, and posterior neck pain several days before admission, and subsequent fever and neurological deterioration on the day of hospitalization. This temporal sequence supports sinusitis as the primary focus, with subsequent progression to meningitis and systemic infection. The patient also presented with eyelid dysfunction and conjunctival injection that improved after endoscopic sinus surgery. Intraoperative findings demonstrated a nasal papilloma with associated bony destruction. Improvement of ocular symptoms after surgery suggests that increased intraorbital pressure contributed to these findings. Sinusitis with orbital extension has been associated with an increased risk of intracranial complications, including cavernous sinus thrombosis, meningitis, and brain abscess [8]. Collectively, these findings indicate a high risk of progression from sinusitis to intracranial infection. Although the exact route of infection cannot be determined, bony destruction may have facilitated local extension through disrupted anatomical barriers or hematogenous dissemination.

The discrepancy between CSF and sinus culture results may be explained by methodological and microbiological factors. CSF cultures were performed using standard aerobic methods only, which limited detection of fastidious anaerobes such as *Dialister pneumosintes*. Although *Staphylococcus aureus* was not isolated from CSF, this finding does not exclude its involvement in meningitis, given an estimated CSF culture sensitivity of approximately 85% [3]. The absence of pathogen detection in CSF may also reflect a low bacterial burden below the detection threshold. Previous reports, including cases associated with brain abscess, have described identification of *D. pneumosintes* exclusively in blood cultures despite negative cultures from the presumed primary site, supporting an oral or nasopharyngeal source [9]. In the present case, failure to detect *D. pneumosintes* in CSF may reflect both methodological limitations and low organism burden during hematogenous dissemination. Additional molecular diagnostics, such as 16 S rRNA gene sequencing, could have strengthened microbiological evidence, but were not performed because of institutional limitations and delayed organism identification. While cases of brain abscess associated with *Dialister pneumosintes* have been reported, acute meningitis due to this organism has not been clearly documented to date, to our knowledge.

Reports of bacteremia caused by *D. pneumosintes* remain limited. A PubMed search identified eight reported cases (Table 2) [2, 9–15], which were analyzed together with the present case. Patient age ranged from 13 to 78 years, with a slight female predominance (male-to-female ratio, 2:7). Most patients had no underlying conditions such as malignancy or immunosuppressive therapy. Seven of nine patients (77.8%) had odontogenic infections or sinusitis as the presumed source of infection. Abscess formation occurred in six patients (66.7%), and septic thrombophlebitis developed in two patients (22.2%). Surgical intervention was required in six cases (66.7%), indicating that antimicrobial therapy alone may be insufficient. In cases with available wound cultures, *D. pneumosintes* was not isolated from the infected sites. Regarding microbiological identification, three cases, including the present case, used MALDI-TOF MS, whereas the remaining six cases used 16 S rRNA gene sequencing. These findings suggest that *D. pneumosintes* is difficult to identify using conventional culture methods and often requires advanced molecular or proteomic techniques.

**Table 2:** Summary of previously reported cases of *Dialister pneumosintes* bacteremia and the present case

Reference No.	Year	Age	Sex	Comorbidities	Presumed source of infection	Associated infections	Diagnostic method	Blood culture results	Other culture results	Antimicrobial therapy	Surgical intervention	Outcome
[9]	2002	17	Male	none	Frontal sinus abscess	Anterior frontal subdural abscess	16S rRNA gene	*D.pneumosintes*	Pus: *Streptococcus anginosus*,* CNS*	AMPC + CAM→CTX + MNZ	Incision and drainage	Recovery
[2]	2006	27	Female	none	Vaginitis	Pyogenic thrombosis	16S rRNA gene	*D.pneumosintes*	none	IMPM + RFP	none	Recovery
[10]	2015	62	Female	Breast cancer	Dental infection Sinusitis	Osteonecrosis of the jaw	16S rRNA gene	*D.pneumosintes*	none	CFPM+LVFX	none	Recovery
[11]	2016	78	Female	none	Dental infection	Apical periodontal abscess	16S rRNA gene	*D.pneumosintes*,* S.exigua*	Pus: *Streptococcus anginosus*	CTRX+CLDM	Incision and drainage	Recovery
[12]	2021	13	Female	Obecity	Sinusitis	Pneumonia, ARDS	MALDI-TOF	*D.pneumosintes*	Tracheal secretion specimen: *Staph.aureus*	PIP/TAZ→MEPM→Amp/sul+CPFX	none	Recovery
[13]	2021	30	Female	none	Dental infection	Neck and Mediastinal Abscess	16S rRNA gene	*D.pneumosintes*	none	MRPM + VCM+FLCZ	Incision and drainage	Recovery
[14]	2022	73	Female	none	Dental infection	Peritonsillar abscess Retropharyngeal abscess Lemierre’s syndrome	16S rRNA gene	*D.pneumosintes*	Pus: Oral commensal bacteria	AMP/SUL + MNZ	Incision and drainage	Recovery
[15]	2023	75	Male	AAA Postoperative	Aortic Esophageal Fissure	Perigraft abscess	MALDI-TOF	*D.pneumosintes*	Puncture fluid: *Streptococcus anginosus*, *Fusobacterium nucleatum*, *Parvimonas micra*, Intraoperative graft༚*Citrobacter koseri*,* Candida albicans*	PIP/TAZ + VCM→PIPC + MNZ+GM	Aortic Graft Removal Surgery	Recovery
	Present Case	65	Female	Breast cancer	Dental infection Sinusitis	Meningitis	MALDI-TOF	*D.pneumosintes*	Pus: *Staph.aureus*	AMPC+CTRX + VCM→CTX	Sinus Surgery	Recovery

Abbreviations: MALDI-TOF MS, matrix-assisted laser desorption/ionization time-of-flight mass spectrometry; ARDS, acute respiratory distress syndrome; AAA, abdominal aortic aneurysm; AMP, ampicillin; AMPC, amoxicillin; CAM, clarithromycin; CTRX, ceftriaxone; CTX, cefotaxime; CLDM, clindamycin; CFPM, cefepime; LVFX, levofloxacin; IPM, imipenem; MEPM, meropenem; MRPM, meropenem; PIP/TAZ, piperacillin/tazobactam; VCM, vancomycin; MNZ, metronidazole; GM, gentamicin

This case report represents an exceptionally rare presentation of *D. pneumosintes* bacteremia complicated by meningitis. Clinically, *D. pneumosintes* often originates from odontogenic or sinonasal infections and demonstrates a propensity for hematogenous spread, frequently resulting in invasive complications such as abscess formation and septic thrombosis. Once identified, prompt systemic evaluation and a treatment strategy incorporating surgical intervention should be considered. With the increasing availability of MALDI-TOF MS and 16S rRNA sequencing, the detection of this fastidious organism has become more feasible. Clinicians should recognize *D. pneumosintes* as a potential but underdiagnosed pathogen in severe anaerobic infections.

### Patient perspective

The patient had no significant prior medical history; however, this hospitalization initiated regular outpatient follow-up. Both the patient and her family expressed gratitude for the resolution of the severe infection. During the hospital stay, a breast cancer diagnosis was made, leading to subsequent surgery and chemotherapy after discharge. The patient has maintained a positive outlook and continues to undergo active treatment.

## Data Availability

Data sharing does not apply to this article as no datasets were generated or analyzed during the study.

## Declarations

### Author information

Dr. Mari Yamamoto is a clinician in the Department of Rheumatology and Nephrology at Chubu Rosai Hospital and a member of the hospital’s Infection Control Team. Her clinical and research interests include case reports and clinical studies in infectious diseases, rheumatology, and nephrology.

## Abbreviations

D. pneumosintes: *Dialister pneumosintes*
MALDI-TOF MS: Matrix-assisted laser desorption ionization time-of-flight mass spectrometry
ICU: Intensive care unit
CT: Computed tomography
CSF: Cerebrospinal fluid
